# Survival benefit of sphingosin‐1‐phosphate and receptors expressions in breast cancer patients

**DOI:** 10.1002/cam4.1609

**Published:** 2018-06-20

**Authors:** Fu‐Ju Lei, Bi‐Hua Cheng, Pei‐Yin Liao, Hsiao‐Ching Wang, Wei‐Chun Chang, Hsueh‐Chou Lai, Juan‐Cheng Yang, Yang‐Chang Wu, Li‐Ching Chu, Wen‐Lung Ma

**Affiliations:** ^1^ Department of Medicine Graduate Institution of Clinical Medical Science Graduate Institute of Biomedical Sciences School of Medicine China Medical University Taichung Taiwan; ^2^ Sex Hormone Research Center Department of Obstetrics and Gynecology Department of Gastroenterology Chinese Medical Research and Development Center China Medical University/Hospital Taichung Taiwan; ^3^ Department of Obstetrics and Gynecology Chang‐Gung Memorial Hospital Chia‐Yi Branch Chia‐Yi Taiwan; ^4^ Research Center for Natural Products and Drug Development Graduate Institute of Natural Products Kaohsiung Medical University Kaohsiung Taiwan; ^5^ Department of Medical Research Kaohsiung Medical University Hospital Kaohsiung Taiwan; ^6^ Department of OBS & GYN BenQ Medical Center Suzhou Jiangsu Province China

**Keywords:** Breast cancer (BCA), Sphingosine‐1‐Phosphate (S1P), S1PR

## Abstract

Sphingosine‐1‐phosphate (S1P) is a bioactive lipid that exerts various pathophysiological functions through binding to its receptor family (S1PRs). Since first report of the breast cancer (BCA) promoting function by S1P production (through the function of sphingosine kinases) and S1P/S1PR signaling, their antagonists have never been successfully progress to clinics after three decades. Taking advantage of bioinformatics linking to gene expression to disease prognosis, we examined the impact of associated genes in BCA patients. We found high gene expressions involved in S1P anabolism suppressed disease progression of patients who are basal cell type BCA or receiving adjuvant therapy. In addition, S1PRs expression also suppressed disease progress of multiple categories of BCA patient progression. This result is contradictory to tumor promoter role of S1P/S1PRs which revealed in the literature. Further examination by directly adding S1P in BCA cells found a cell growth suppression function, which act via the expression of S1PR1. In conclusion, our study is the first evidence claiming a survival benefit function of S1P/S1PR signaling in BCA patients, which might explain the obstacle of relative antagonist apply in clinics.

## 1 INTRODUCTION

Sphingosine‐1‐phosphate (S1P) is a bioactive sphingolipid metabolite involved in many pathophysiological processes.^1^, ^2^, ^3^, ^4^ The intracellular level of S1P as a secondary messenger 5 is regulated by several enzymes including anabolism and catabolism. For anabolism, such as sphingosine kinases 1 and 2 (SPHK1, SPHK2) that are responsible for S1P synthesis. And S1P lyase1 (SGPL1) and S1P phosphatase 1 and 2 (SGPP1, SGPP2) that mediated S1P catabolism.^5^, ^6^, ^7^ Besides, lipid phosphate phosphatases (LPP1/2/3) regulates lipid dephosphorylation including hydrolyzing S1P. In addition, S1P could be exported to extracellular environment by the ATP‐binding cassette transporters (ABCA1, ABCC1).^5^, ^8^ Recent studies indicated spinster homolog 2 (Spns2) expressed on endothelial cells (ECs) function as S1P transporter.^9^, ^10^ In addition, studies showed that red blood cells and platelets requires the major facilitator superfamily transporter 2b (Mfsd2b) to export S1P 11, 12 and maintained S1P level in blood stream. The function of S1P is go through its receptors S1PRs (S1PR1‐5) which belongs to the G‐protein coupled receptor (GPCR) family 2 and each receptor, respectively, involved in cell growth, apoptosis, proliferation, angiogenesis, chemo resistance, or vascular stability.^5^, ^13^, ^14^, ^15^ It is also a chemotactic factor for immune cell trafficking 5, 16, 17 and a microenvironmental regulator for cancer development.^18^ The growth rate of breast cancer cells was found to be decreased in SPHK1 or SPHK2 deficient mice.^19^ Based on these observations, S1P promote breast cancer (BCA) development was hypothesized. The utilization of online cDNA microarray databases meta‐analysis that predicts the outcome with appropriately powered cohorts and provides feasible, unbiased approach to analyze genes in cancer progression. There are online databases to validate the importance of gene expressions in BCA patient survival (http://kmplot.com/analysis/index.php?p=service&cancer=breast). Beneficiary from the advances of bioinformatics, using the web‐based genome‐scale gene expression survival analyzer followed with pathway weighting algorithm, we are able to evaluate gene clusters/pathways‐of‐genes in cancer patient prognosis in a hypothesis‐driven basis.^20^ To test our hypothesis, we performed Kaplan Meier plotter to investigate the role of S1P in BCA patient survival (http://kmplot.com/analysis/index.php?p=service&cancer=breast) and the association between S1P metabolism and BCA progression. The role of S1P receptors (S1PRs) in BCA development was also investigated.

## 2 MATERIALS AND METHODS

### 2.1 Kaplan‐Meier plot for evaluating the impact of genes in BCA patient survival

Data mining was performed using Kaplan‐Meier Plotter (http://kmplot.com/analysis/index.php?p=service&cancer=breast).^21^ The following genes that may play a role in breast cancer 10‐year relapse free survival (RFS) were assessed *SPHK1* (219257_s_at), *SPHK2* (209857_s_at), *SGPP1* (208381_s_at), SGPP2 (226560_at), LPP1 (210946_at), LPP2 (209529_at), LPP3 (212230_at), *SGPL*1 (223391_at), *S1PR*1 (204642_at), *S1PR*2 (208537_at), *S1PR*3 (228176_at), *S1PR*4 (206437_at), and *S1PR*5 (221417_x_at). Databases of the following cohorts were analyzed: nonclassified BCA (n = 1660); ER +/− (n = 695/313); PR +/− (n = 489/372); HER2 +/− (n = 150/635); lymph node metastasis positive and negative (n = 724/496); grade 1, grade 2, and 3 (n = 108/227/444); and intrinsic subtypes including basal (n = 339), luminal A (n = 841), and luminal B (n = 407): Tp53 mutant or wild type (n = 132/82); with systemically treatment (n = 751); include or exclude endocrine therapy or Tamoxifen only (n = 335/275/161); and with or without chemotherapy (n = 255/243). According to the gene expression we use median to divide the patients into two groups, low and high expression.

### 2.2 Determination of Hazard Ratios (HR score)

The following formula was used to calculate HR scores^20^:

$$\begin{array}{cc} \text{HR score}= & \text{Avg. HR score of gene sets}=\\ & \frac{\sum \left({\text{HR}}_{n}-1\right)\times \left(-{\text{log}}_{10}\left(P\;\text{value}\right)\right)}{n}\times 100 \end{array}$$

### 2.3 Cell culture, reagents, and chemicals

The human breast cancer cell line MCF‐7 and human triple negative breast cancer (TNBC) cell lines MDA‐MB‐231 were purchased from the Food Industry Research and Development Institute in Taiwan. Cells were grown in Dulbecco's Modified Eagle Medium (GIBCO, USA) with 10% fetal bovine serum (GIBCO, CA,USA) and 1% penicillin‐streptomycin solution (CORNING, NY, USA) at 37°C and 5% CO_2_. Sphingosine‐1‐phosphate was obtained commercially (Sigma‐Aldrich, MO, USA) and dissolved in methanol.

### 2.4 Cell colony formation assay

Effects of S1P on the growth of various types of BCA cells were assessed by colony formation assay. 500 each of MCF‐7 and MDA‐MB 231 cells were seeded separately in wells of a 6‐well plate. S1P was added to the cultures at 0, 2, 4, 6, 8, 10 μmol/L 24 hours after seeding. After 2 weeks of incubation, culture media were removed, and cells were washed with PBS once. The cells in each well were then fixed with 1 mL of 4% formaldehyde for 30 minutes. After removal of the formaldehyde solution, cells in each well were stained with 1.5 mL of 2% crystal violet for 30‐60 minutes, and colony number of each cell type was counted.

### 2.5 Lentiviral production and infection

To investigate the role of S1P in the growth of BCA cells, miRNAs targeting the genes encoding its receptors S1PR1 and S1PR2 were introduced into cells using lentiviral vectors as described previously. Lentiviral plasmid vectors containing shRNA genes targeting S1PR1 (TRCN0000221119; shS1PR1 sequence: CCGGCCCATGTGAAAGCGTCTCTTTCTCGAGAAAGAGACGCTTTCACATGGGTTTTT) and S1PR2 (TRCN0000356882; shS1PR2 sequence: CCGGCAAGGTCCAGGAACACTATAACTCGAGTTATAGTGTTCCTGGACCTTGTTTTTG) and the control plasmid (pLKO.1‐shLuciferase) were obtained from the National RNAi Core Facility, Academia Sinica, Taipei, Taiwan. To produce lentiviral vectors, HEK293T cells were transfected with each of these plasmid together with packaging plasmid (psPAX2) and envelope plasmid (pMD2.G) (Addgene, MA, USA) at a ratio of 2:1:1 by liposome in 3 mL of serum free DMEM (GIBCO, CA, USA). The culture medium was then replaced by 10 mL of fresh DMEM with 10% FBS 4‐6 hours after transfection. The culture supernatants containing recombinant lentiviruses were harvested 48 hours after incubation and filtered through a 0.45 μm filter to remove dead cells and debris. BCA cells at 40‐50% confluence were washed with PBS once and then incubated with 5 mL culture medium containing lentiviral vectors and 8 μg/mL of polybrene (Sigma‐Aldrich, MO, USA) for 24‐48 hours. Cells that were then incubated in fresh DMEM containing puromycin (1 μg/mL) to eliminate those that were not infected with lentivirus.

### 2.6 RT‐PCR

To evaluate S1PR1 and S1PR2 knockdown, RT‐PCR was performed as described. Briefly, RNAs were isolated from lentivirus infected cells (80‐90% confluence) using the Trizol reagent (Invitrogen, CA, USA) and reverse transcribed to cDNAs using the PrimeScript TM RT reagent kit (TAKARA Bio Inc., Kyoto, Japan). The real‐time PCR was performed using the KAPA TM SYBR FAST One‐Step qRT‐PCR Kit (KAPABIOSYSTEMS, MA, USA) with the following primers: S1PR1 (forward 5′‐CGAGAGCAC TATGCAGTCAG‐3′, reverse 5′‐CGATGAGTGATCCAGGCTTT‐3′), S1PR2 (forward 5′‐AGGTCGTCTCCTGCGTTTC, reverse 5′‐GCCGGCCTAGCCAGTTCT‐3′). The KAPA TM SYBR FAST One‐Step qRT‐PCR Kit (KAPABIOSYSTEMS, MA, USA) and temperature condition were used according to the manufacturers’ instructions.

### 2.7 Western blot

To measure S1PR1 and S1PR2 knockdown effect in protein level, western blot was performed. Briefly, protein separation through SDS‐PAGE by gel electrophoresis. Gel percentage was 8% and then transferred to a PVDF membrane (Millipore, MA, USA). The membrane was then incubated with the target antibodies, S1PR1/2 (R12‐3478/R12‐2725, AssaybioTech, CA, USA). After incubation, ECL reagent (Millipore, MA, USA) was applied to the membranes, and used Chemidoc XRS+ (BioRad) with a charge‐coupled device (CCD) camera to capture the signals.

### 2.8 Statistics

Significant difference between the experimental group and the control group was determined using student's t‐test. Results were expressed as mean ± SEM.

## 3 RESULT

### 3.1 S1P producing enzyme expression attenuates breast cancer development

**Figure 1:**
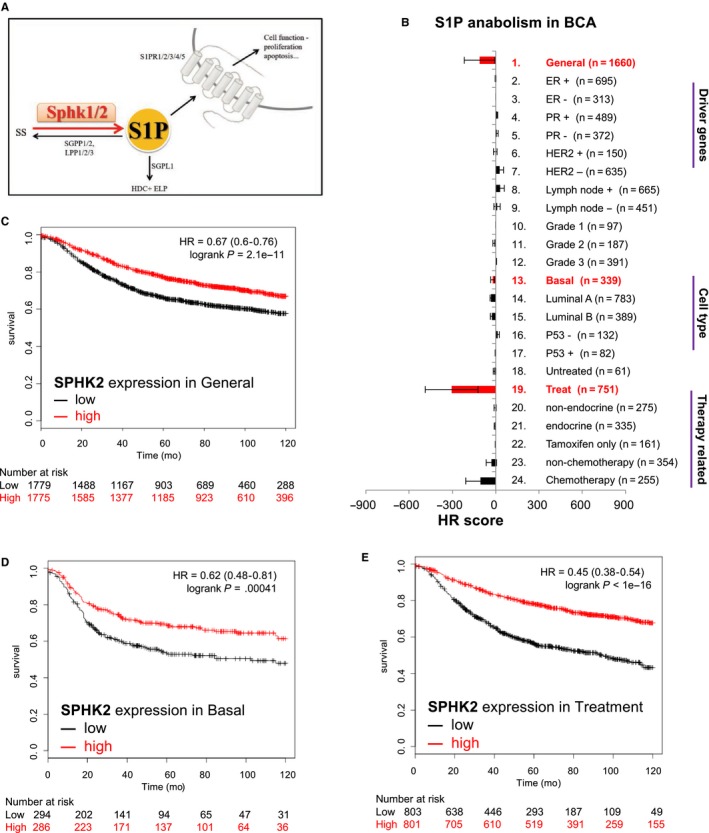
S1P anabolism attenuate prognosis for BCA patients. A, The illustration of S1P production and the involved enzyme (Sphk1 and Sphk2). SS: sphingosine; SPHK 1/2: sphingosine kinase 1/2; SGPP1/2: Sphingosine‐1‐phosphate phosphatase 1/2; S1P: Sphingosine‐1‐phosphate; SGPL1: Sphingosine‐1‐phosphate lyase1; LPP 1/2/3: lipid phosphate phosphatases 1/2/3; HDC: Hexadecenal; ELP: Ethanolamine phosphate; S1PR1/2/3/4/5: Sphingosine‐1‐phosphate receptor 12/3/4/5. B, HR score to evaluate the impact of S1P anabolism in BCA prognosis. The items on the right are the patient classification for KM plotter analyses. C‐E, Kaplan‐Meier plot survival analysis of SPHK2 in overall patients (C), basal cell type (D), and patients with adjuvant therapy (E). In *X*‐axis is survival of observe patients, *Y*‐axis is observed time for 10 years. The words low and high means the target gene expression was low or high in patients. Number at risk means the observe number of patients

The effects of S1P anabolic enzymes (SPHK 1 and SPHK 2) (Figure 1A) on BCA progression were first investigated. Analyses of databases of various BCA cohorts using KM Plotter with a defined prognosis threshold as 10‐years recurrence risks (relapse‐free survival; RFS) 22 for evaluating various patient classifications. The results exhibited that little influence on most classifications of BCA patients (Figure 1B). SPHK1 had no significant effect on 10‐year relapse free survival (data not shown). However, SPHK2 expression was found to have a positive impact in patients with nonclassified (Figure 1C, HR = 0.67, *P*‐value = 2.1e‐11) or basal cell type BCA (Figure 1D, HR = 0.62, *P*‐value = 4.1e‐4) and in those received adjuvant therapy (Figure 1E, HR = 0.45, *P*‐value <1e‐16). Those data indicating SPHK2 is the determining enzyme to suppress adjuvant treatment BCA patient progression.

### 3.2 S1P catabolism plays redundant roles in breast cancer progression

**Figure 2:**
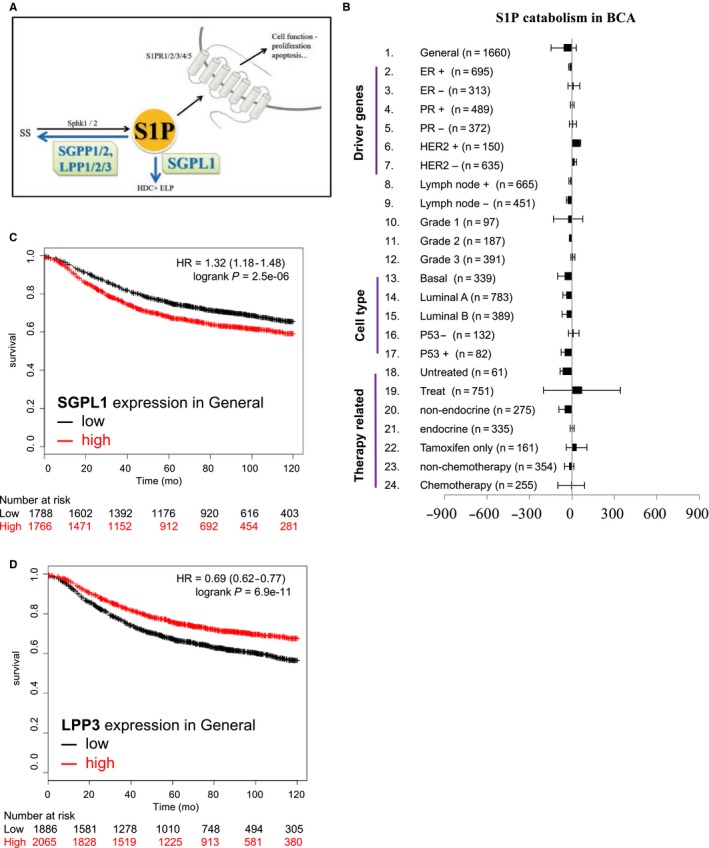
S1P catabolism did not affect prognosis for BCA patients. A, The illustration of S1P catabolism and the involved enzyme (SGPP1/2, SGPL1 and LPP 1/2/3). B, HR score to evaluate the impact of S1P catabolism in BCA prognosis. The items on the left is the patient classification for KM plotter analyses. C and D, Kaplan‐Meier plot survival analysis of SGPL1 in overall patients (C) and LPP3 in general parameter (D)

As the dynamic homeostasis of S1P is also related to its catabolism (Figure 2A), the roles of enzymes (SGPP1/2, SGPL1, and LPP1/2/3) responsible for its degradation in BCA progression were examined. Results showed that SGPL1 expression significantly negative‐correlated with BCA progression in patients of the nonclassified (Figure 2C, HR = 1.32, *P*‐value = 2.5e‐06). However, LPP3 showed positive correlation with BCA progression in general condition (Figure 2D, HR = 0.69, *P*‐value = 6.5e‐11). In brief, S1P degradation may not be one of the factors affecting the progression of breast cancer.

### 3.3 S1P receptors expression attenuates breast cancer progression

**Figure 3:**
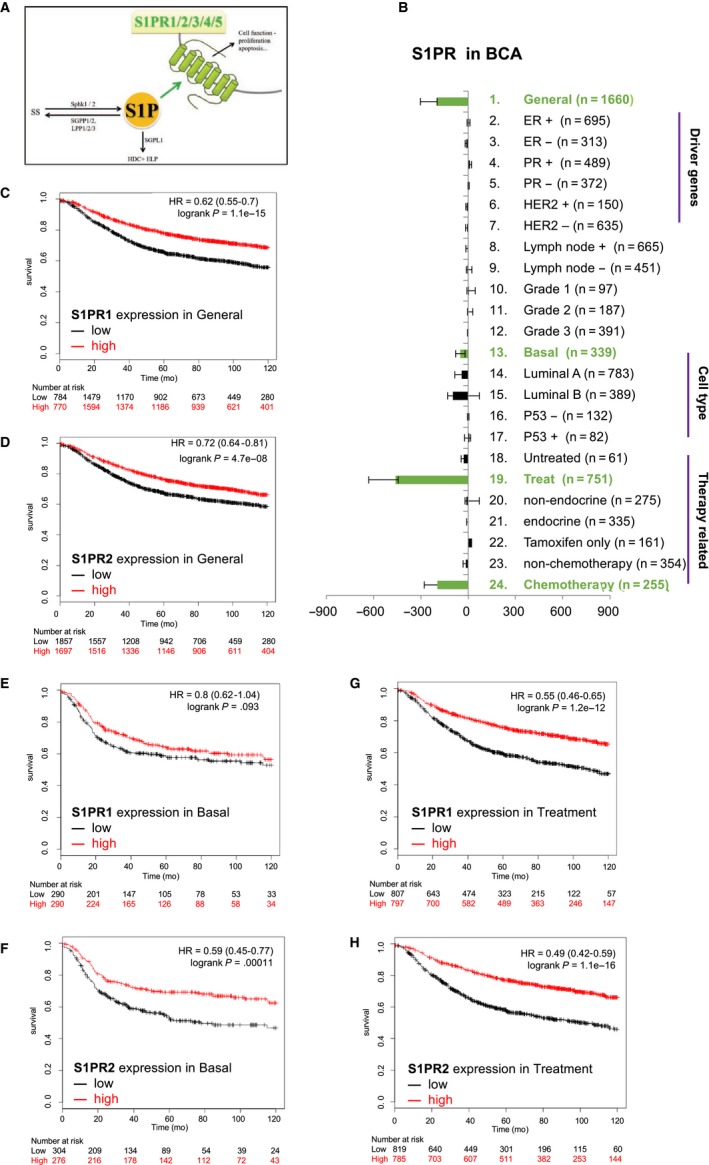
S1P receptors attenuate prognosis for BCA patients. A, The illustration of S1P effective receptors (S1PR1,2,3,4,5). B, HR score to evaluate the impact of S1P catabolism in BCA prognosis. The items listed on the right‐hand side is the patient classification for KM plotter analyses. C,E,G, Kaplan‐Meier plot survival analysis of S1PR1 in overall patients (C), basal cell type (E), and patients with adjuvant therapies (G). D,F,H, Kaplan‐Meier plot survival analysis of S1PR2 in overall patients (D), basal cell type (F), and patients with adjuvant therapy (H)

As S1P exerts its biological functions through S1P receptors (S1PRs), the association of S1PR expression and BCA progression was investigated (Figure 3A; S1PR1‐5) Results showed that the expression of S1PRs had a positive impact on BCA patients (Figure 3B). Individual dissection of S1PRs revealed that S1PR1 expression levels were found to suppressed the progression of tumors in patients with nonclassified BCA (Figure 3C; HR = 0.62 with *P*‐value = 1.1e‐15) and in those received treatments (Figure 3G; HR = 0.55 with *P*‐value = 1.2e‐11). However, S1PR1 expression had no significant effects on patients with basal cell type BCA (Figure 3E; HR‐0.8 with *P*‐value = .093). S1PR2 expression was found to have a positive impact on patients with nonclassified (Figure 3D, HR = 0.72, *P*‐value = 4.7e‐08) or basal cell type (Figure 3F, HR = 0.59, *P*‐value = 1.1e‐04) BCA and on those received treatments (Figure 3H; HR = 0.49 with *P*‐value = 1.1e‐16).

### 3.4 S1P suppresses breast cancer cell growth through S1PR1 in vitro

**Figure 4:**
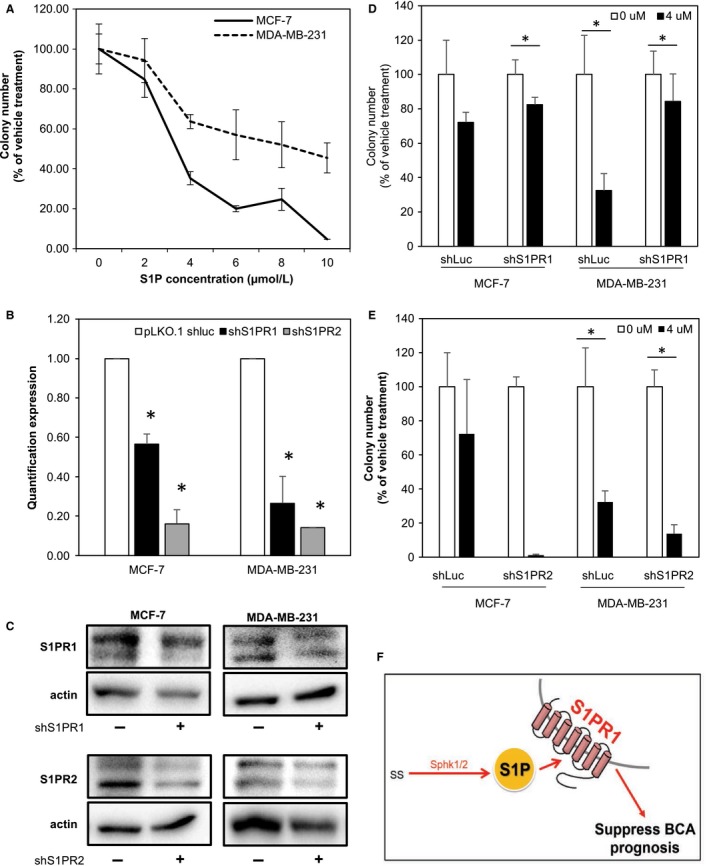
S1P inhibit cell colony through S1PR1. (A) S1P suppress BCA cells colony formatting ability. MCF7 was estrogen sensitive cells, MDA‐MB‐231 was triple negative breast cancer cell. The shRNA efficiency of S1PR1 and S1PR2 expression in quantification RCR and western blot (B, C). (D) Colony formation of knockdown S1P receptor 1 in MCF‐7 and MDA‐MB‐231 cells. S1P inhibitory effect was abolished when knockdown S1PR1 in MDA‐MB‐231 cell. E. Colony formation of knockdown S1P receptor 2 in MCF‐7 and MDA‐MB‐231 cells. The * indicating *P*‐value <.05. The data shown were the mean value of reproducible three biological repeats. (F) The carton shows S1P suppressed breast cancer prognosis through S1PR1. High abundance of S1P suppresses breast cancer prognosis (red arrows); on the contrary, low level of S1P promotes breast cancer prognosis (blue arrows)

To confirm the inhibitory effect of S1P on BCA progression, the effect of S1P on the growth of BCA cells was investigated in vitro. To achieve the goal, MCF‐7 (hormone sensitive) and MDA‐MB‐231 (triple negative breast cancer, TNBC) cells were treated with 0‐10 μmol/L of S1P for 2 weeks and then evaluated for growth by colony formation assay. Results showed that S1P inhibited the proliferation of these cells in a dose‐dependent manner (Figure 4A), suggesting that SIP can inhibit the growth of both hormone sensitive and hormone insensitive BCA cells.

To confirm the suppressive effect of S1P on the growth of BCA cells, the expression of S1PR1 or S1PR2 in MCF‐7 and MDA‐MB‐231 cells was knocked down by introducing lentivirus carrying S1PR1 or S1PR2 shRNA gene. Results showed that the expression of both S1PR1 and S1PR2 was successfully knocked down by more than 85% (Figure 4B,C) These S1PR1 and S1PR2 knockdown cells were then treated with 0 or 4 μmol/L of S1P for 2 weeks and then assayed for colony formation. As shown in Figure 4D, S1PR1 knockdown MDA‐MB‐231 yielded poor inhibition than vehicle cell while treat with S1P; yet, we did not observe S1PR1 knockdown reverse S1P effect in MCF‐7 cells. In Figure 4E, knockdown S1PR2 did not influence the S1P inhibition in MDA‐MB‐231 cell, moreover, MCF‐7 showed more dramatic suppression in S1P treatment. These data suggest that S1P inhibit BCA cells partially go through S1PR1 and irrelevant to S1PR2 cells.

## 4 DISCUSSION

### 4.1 SPHK/S1P/S1PR signaling in BCA cells

Our data showed that S1P can suppress BCA prognosis and this effect was partially through S1PR1 expression (Figure 4E). This result is contradictory with those of previous studies.^6^, ^19^, ^23^, ^24^ We discuss this inconsistency in the following.

Ceramide and sphingosine are the precursors of S1P. Literatures indicated that ceramide and sphingosine involved in cell death, apoptosis, and anti‐proliferation.^25^, ^26^, ^27^ SGPP1 is the enzyme revert S1P to ceramide/sphingosine, add‐in S1P might increase ceramide/sphingosine level from SGPP1. However, the results from Le Stunff et al^28^ showed that increase in S1P did not upregulate ceramide level unless overexpressed SGPP1 expression. Since that, ceramide and sphingosine from S1P catabolism might not contributor to the S1P‐mediated cancer inhibitory effect.

SPHK1 is an enzyme responsible for S1P production and is involved in many cellular processes, such as promoting cellular proliferation and stimulating cell survival and metastasis.^2^, ^3^, ^4^ SPHK1 mainly express in the cytosol and needed to be translocated to the membrane while be activated. SPHK1 increases S1P levels in the local environment and induced proliferation signaling.^29^ On the other hand, SPHK2 presented and form S1P in the nucleus.^30^ In our study, we added S1P in vitro which increases extracellular S1P concentration to mimic Sphk1 pathway to induce proliferation signals. Since we discovered the S1P cancer growth inhibition effect, we excluded the growth promoting mechanisms as the signaling route. According to the literatures, Insulin‐like growth factors (IGFs) stimulated cell proliferation by activated MAPK, Akt 31 and S1P have been found to inhibit keratinocyte proliferation through inhibit Akt/PKB pathway induced by Insulin‐like growth factor I (IGF‐I).^32^ In the cancer condition, triple negative breast cancer was reported to express high level of IGF‐I.^31^, ^33^ Therefore, S1P/S1PR1 signaling might downregulate IGF‐1/Akt/PKB axis to suppress cell proliferation.

Results of previous study indicated that overexpression of SPHK1 in MDA‐MB‐231 resulted in a decrease in sphingosine concentration and knockdown of SPHK1 with shRNA increased sphingosine level.^23^ The function of SPHK1 was suggested to upregulate sphingosine levels and influence cell proliferation. Besides, the study in 2008 also showed SPHK1 correlated with poor prognosis in ER‐negative breast cancer.^34^ Moreover, in 2010 Watson et al^35^ found that high expression of SPHK1 and ERK‐1/2 is associated with shorter recurrence time in ER positive patients. Besides, high level of S1PR1 and ERK1/2 or high expression of S1PR3 and ERK1/2 also indicated the association with shorter recurrence times. These studies illustrated that overexpression of SPHK1 has been shown to increase cancer progression. Ohotski et al^36^ also indicated that nuclear localization of sphingosine kinase 1 and S1PR2 played an important role in breast cancer prognosis. Yet it is still unknown whether this effect is directly related to S1P level in these studies.

Previous study has been shown that S1P stimulates OVCA invasion at low concentration (0.5 μmol/L), but inhibits its invasion at high concentration (20 μmol/L).^37^ In our study, the minimum dosage required to inhibit colony formation of BCA cells was 4 μmol/L (Figure 4A). Literatures showed that the levels of S1P in the ascitic fluids ranged from 0.42‐2.17 μmol/L,^38^ and the serum S1P concentration in healthy individual is less than 1 μmol/L.^5^ Referencing with the S1P concentration and our observations, we speculated that local S1P concentration may suppresses BCA cell proliferation. Recently, Nagahashi et al^39^ used the breast cancer patients tissue to measured sphingolipid level and found S1P level was higher in tumor tissue than normal tissue. However, they did not establish the relationship with S1P level, disease progression and patient survival.

S1P signal transduction is through binding to a family of five G protein‐coupled receptors (GPCRs). S1P receptors are divided into five subtypes: S1PR1, S1PR2, S1PR3, S1PR4, and S1PR5, and S1PRs are differentially coupled to heterotrimeric G proteins and Rac or Rho to regulate various effectors, such as MAPKs.^1^, ^14^, ^40^, ^41^ Previous studies have shown that S1P‐SIPRs signaling can enhance cell proliferation and migration. The high S1PR1 expression in glioblastoma patients was positively associated with favorable survival.^42^ However, S1PR2 signaling negatively regulates tumor angiogenesis and tumor growth in vivo.^43^ S1PR2 is also involved in the reduction of platelet‐derived growth factor mediated‐cell motility and proliferation.^44^ A knockdown in the expression of S1PR2 by shRNA in satellite cells resulted in a twofold increase in cell migration.^45^ As mentioned above, we observed that S1P can enhance BCA cancer cell growth when it binds to S1PR1 and in the KM plotter analysis, S1PR2 expression was found to have a better survival benefit than S1PR1 expression. There also a study investigated other receptor, such as S1PR4, and the authors suggested S1PR4 related to ER‐negative breast cancer progression.^46^ Since our KM plotter did not showed the significant result in S1PR4, we do not rule out the possibility that different S1PR subtype will have diverse function in different type of breast cancer.

Another study to analyze sphingolipid level in tumor tissue of breast patients and demonstrated high expression of pSPHK1 is associated with higher levels of S1P, which in term is associated with lymphatic metastasis in breast cancer.^47^ Some references providing clinical evidence to show the S1P axis involved in the therapies resistant of tumors. In Katsuta et al^48^ study, it suggested that the expression of sphingosine kinase 1 tended to be higher in doxorubicin‐resistant human cancer and cell lines. In Nagahashi et al^49^ recent study, they used FTY720 to target SPHK1/S1P/S1PR1 axis and suggested that this pathway is associated with obesity‐related inflammation and breast cancer metastasis. Since our KM plotter results did not show the relationship with metastasis and we did not investigate the drug resistant effect, we were optimistic about the above studies that S1P might played different role in different tumor progression.

### 4.2 S1PR as a target for BCA treatment

S1P axis was initially found to play important roles in lymphocyte egress from lymphoid tissue. It means that targeting S1P may have immunosuppressive function. This possibility has been investigated in organs transplantation and autoimmune diseases. Although the effects of targeting S1P axis for cancer treatment are still preliminary, a few clinical trials have explored the anti‐tumor effects of S1P related modulators. The currently known drug targeting S1P is FTY720 (Fingolimod), which is an analog of sphingosine and functions as S1PR agonist. However, while FTY720 phosphorylated by SPHK2 to form FTY720‐phosphate (FTY720‐p) was suggested to a functional antagonist of S1PR1 through binding to S1PR1 and induces internalization and degradation, leading to sequestration of thymocytes and lymphocytes from secondary lymphoid organs thereby reducing inflammation.^2^, ^5^, ^50^, ^51^ FTY720 demonstrates anti‐cancer properties and may have a potential in cancer treatment.^52^, ^53^ There are many in vitro and in vivo studies demonstrated the growth arrest and apoptosis‐inducing ability of FTY720. The anti‐cancer ability of FTY720 may be through the inhibition of sphingosine kinase 1. For example, the cisplatin‐resistant melanoma cells treated with FTY720 might activated p53‐independent caspase and caused SPHK1 degradation and might inhibit the Akt/mTOR pathway.^54^ In another way, not all relevant features of FTY720 are helpful for cancer treatment. Nagaoka et al^52^ showed that FTY720‐P did not decrease the viability of BCA cells, suggesting that the anticancer effect of FTY720 was not mediated by its phosphorylate form. Noticeably, they also found that S1P can suppress the growth of BCA as we did in this study. KRP‐203 is a kind of S1PR1 selective agonists. It has been demonstrated to regulate both T and B cells and improve the outcome of atherosclerosis disease.^55^ SEW2871 is also a selective S1PR1 agonist and has been used to treat acute kidney injury and ischemia‐reperfusion injury in mice.^56^, ^57^ SEW2871 binds to S1PR1 causing its internalization and recycling. Those S1PR1 agonists could be candidates for the further investigation of the role of S1P/S1PR in cancer progression. Although we did not observe effect of S1P‐S1PR2 binding in the inhibition of colony formation of BCA cells, results of our bioinformatic analyses showed a positive correlation between S1PR2 expression and BCA progression. There is a compound designated CYM‐5520 58 has been shown to more selective for activating S1PR2 and may be suitable for BCA treatment. Although these compounds are currently available only for research, they may become effective regimens for BCA treatment, based on our findings in this study.

## 5 CONCLUSIONS

In this study, we found a cancer suppressive function of S1P through S1PR1. As this finding is contradictory with those of previous studies, further investigations are needed to develop S1PR agonists for cancer therapy.

## CONFLICT OF INTEREST

All authors in this study claim no interest of conflict.
